# Synergies between assisted reproduction technologies and functional genomics

**DOI:** 10.1186/s12711-016-0231-z

**Published:** 2016-08-01

**Authors:** Pasqualino Loi, Paola Toschi, Federica Zacchini, Grazyna Ptak, Pier A. Scapolo, Emanuele Capra, Alessandra Stella, Paolo Ajmone Marsan, John L. Williams

**Affiliations:** 1 Laboratory of Embryology, Faculty of Veterinary Medicine, University of Teramo, Teramo, Italy; 2 Institute of Genetics and Animal Breeding of the Polish Academy of Sciences, Postepu 36A, Jastrzębiec, 05-552 Magdalenka, Poland; 3 Istituto di Zootecnica, Università Cattolica del Sacro Cuore, Piacenza, Italy; 4 Proteomic and Nutrigenomic Research Center - PRONUTRIGEN, Università Cattolica del Sacro Cuore, Piacenza, Italy; 5 Institute of Agricultural Biology and Biotechnology, National Research Council, 26900 Lodi, Italy; 6 Davies Research Centre, School of Animal and Veterinary Sciences, University of Adelaide, Roseworthy, SA 5371 Australia; 7 National Research Institute of Animal Production 1, Krakowska Street, 32-083 Balice n/Kraków, Poland

## Abstract

This review, is a synopsis of advanced reproductive technologies in farm animals, including the discussion of their limiting factors as revealed by the study of offspring derived from embryos produced in vitro and through cloning. These studies show that the problems of epigenetic mis-programming, which were reported in the initial stages of assisted reproduction, still persist. The importance of whole-genome analyses, including the methylome and transcriptome, in improving embryo biotechnologies in farm animals, are discussed. Genome editing approaches for the improvement of economically-relevant traits in farm animals are also described. Efficient farm animal embryo biotechnologies, including cloning and the most recent technologies such as genome editing, will effectively complement the latest strategies to accelerate genetic improvement of farm animals.

## Background

### Brief history of reproduction biotechnologies

**Fig. 1:**
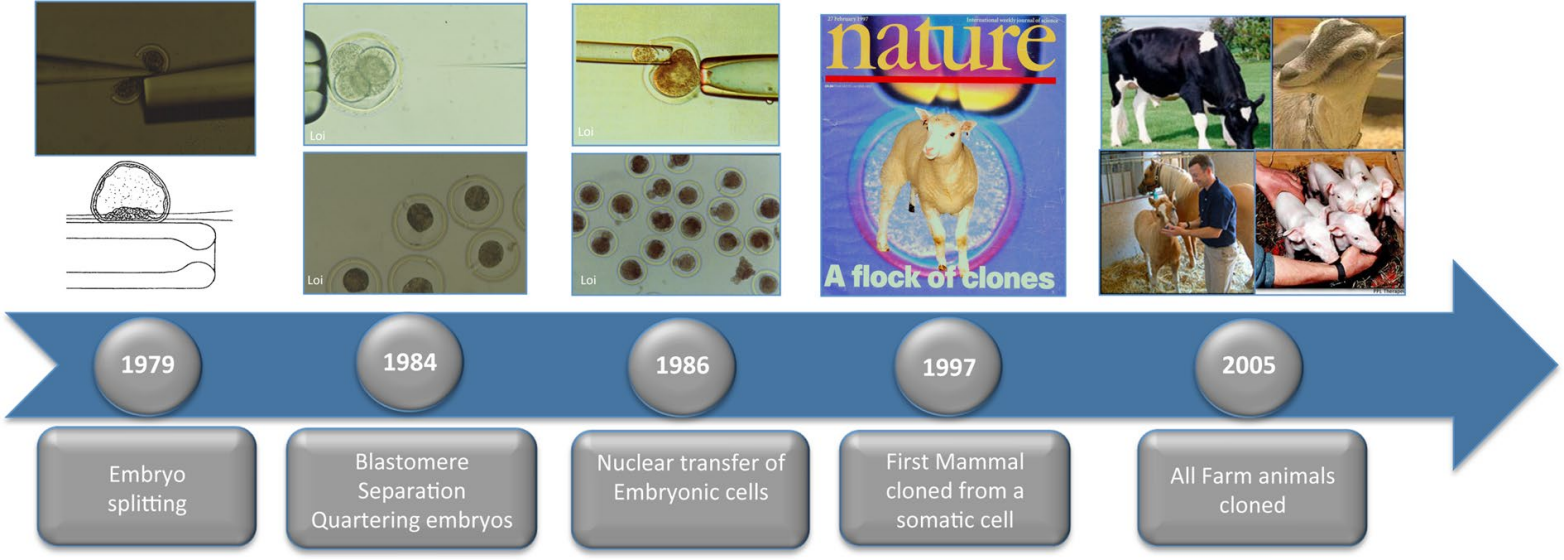
Timeline of the development of reproductive biotechnologies in farm animals

**Fig. 2:**
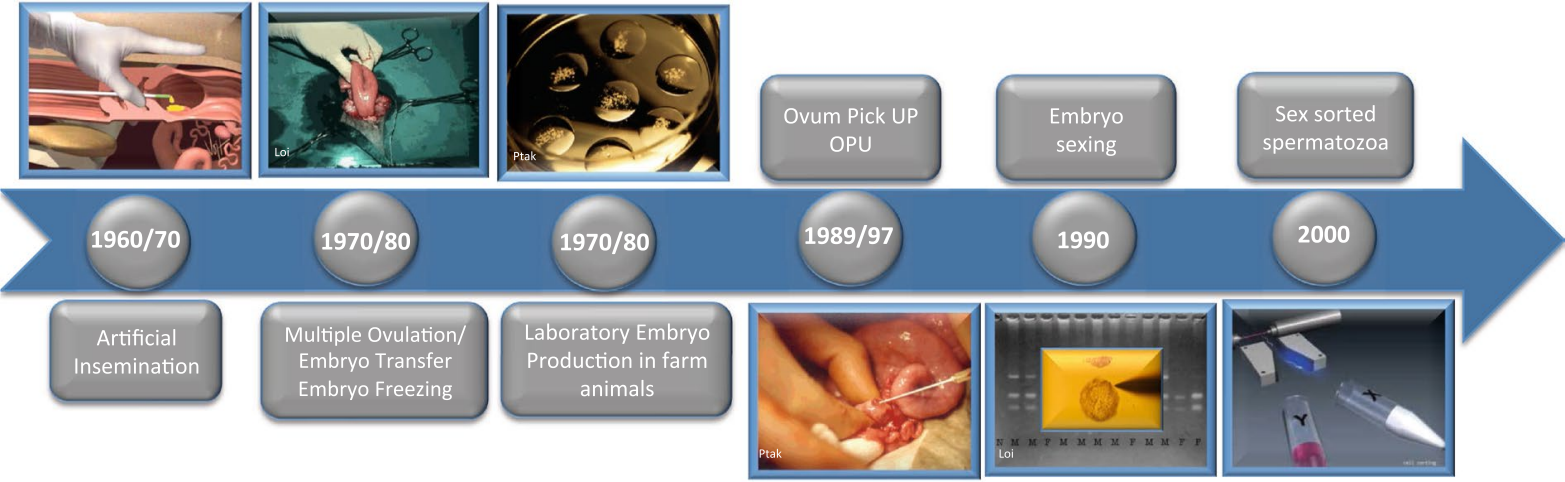
Timeline of embryo multiplication technologies in farm animals

Artificial insemination (AI) was first performed in dogs by Spallanzani in 1784, but this procedure only became routine in the mid 1900s when it was applied to a wide range of species. AI has had the greatest impact in dairy cattle breeding. While AI is an effective way to disseminate the genetics of sires, the portfolio of embryo biotechnologies now available has expanded the opportunities to increase selection pressure on the female side. In the 1980s and 1990s, major advances were made in multiple ovulation and embryo transfer (MOET), ovum pick up (OPU) [1–3] and in vitro embryo production and freezing [4–7] (Fig. 1). In addition, embryo multiplication procedures, including embryo splitting [8–10] and cloning by nuclear transfer of embryonic cells, have been developed [11–14]. The long sought after goal of multiplying unique genotypes culminated with the production of the first mammal cloned by nuclear transfer from a somatic cell [15–17] (Fig. 2). This triggered a negative reaction from those who feared potential applications of this technology.

Paradoxically, although reproduction specialists and quantitative geneticists share the common goal of achieving genetic improvement, these two groups of scientists do not collaborate on a regular basis. With the exception of MOET, embryo biotechnologies have not been used extensively in breeding programs. Fuelled by advances in DNA sequencing and genotyping techniques and by falling costs, genomic selection, first mooted in 2001 [18], is now possible. This approach provides the opportunity to genetically select embryos and increase the use of in vitro methods to accelerate genetic improvement (see companion reviews of the ISAFG meeting in this issue).

**Fig. 3:**
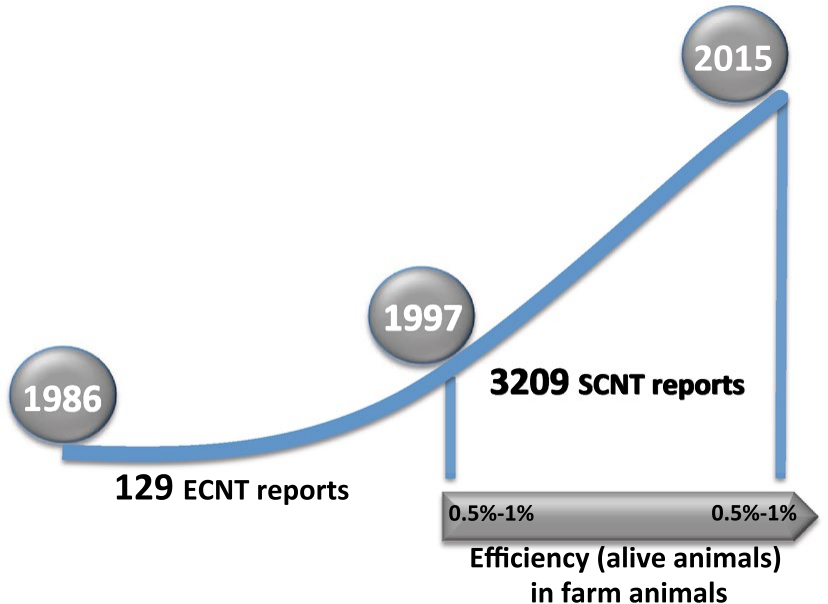
Trend in publications on cloning using embryonic cells nuclear transfer (ECNT) and later somatic cell nuclear transfer (SCNT). Overall efficiency since the production of the first cloned sheep Dolly

In vitro embryo production is not yet reliable, and still essentially uses protocols that were developed in the 1990s. However, we are beginning to understand the molecular mechanisms that are at play during development, and the factors that need to be improved. The techniques used for in vitro production and culture of embryos can lead to alterations in epigenetic programming, e.g. modifications of the DNA methylation patterns. These changes have an effect on the expression of imprinted genes and hence on developmental abnormalities, resulting in foetal overgrowth [19] and failure [20, 21]. Early studies on cloning showed that synchronisation of the nucleus from donor cells with that of the recipient is necessary to sustain the development of a viable embryo [15], most probably through epigenetic reprogramming. However, current cloning techniques are not significantly better than those of the initial cloning reports [22, 23] (Fig. 3), and the success rate of producing viable offspring is still less than 1 %. With this low success rate, cloning cannot be used in programs for the genetic improvement of farm animals, but is an approach that can be used for research. Cloning may also be used to create “back-up” individuals of animals with unique genetic features [24].

Advances are, however, being made in the field of oocyte recovery, culture and in vitro fertilisation. For example, young lambs of 3 to 4 weeks of age have a large number of follicles and, after stimulation, up to 100 follicles can be routinely produced, which yield 60 to 70 oocytes by OPU. More than 50 % of these oocytes are competent and will develop into blastocysts following in vitro fertilisation [25]. This process of juvenile in vitro fertilisation and embryo transfer (JIVET) allows large numbers of full-sibs to be obtained from lambs at a few weeks of age, thus greatly reducing the generation period. Improvements in this approach could accelerate genetic progress by producing large numbers of lambs from elite ewes. JIVET can also be used to produce large numbers of embryos for genetic manipulation, as discussed below.

### New technologies: from SNPs to whole-genome sequencing

The last 10 to 15 years have witnessed rapid advances in approaches for the analyses of DNA sequence and structure. Genome sequence data from next-generation sequencing platforms have identified large numbers of single nucleotide polymorphisms (SNPs), high throughput genotyping platforms have made SNPs the most widespread and efficiently genotyped genetic markers. High-density SNP data can be used in genomic selection (GS) [26] and in genome-wide association studies (GWAS) to identify quantitative trait loci (QTL) for production related traits, such as meat and milk composition, fertility or disease response (see [27, 28]). These data can be used for both in vivo and in vitro breeding applications. SNP data can also be analysed to detect chromosomal regions with loss of heterozygosity (LOH). LOH haplotypes have a significantly reduced frequency, or are absent in the homozygous state. Such haplotypes are likely to contain recessive, deleterious or lethal genetic variants [29–33]. The combined use of SNPs, exome and whole-genome sequencing data from more than 25,000 Fleckvieh cattle led to the detection of four LOH regions [34]. Combining LOH information with large whole-genome sequence datasets, such as the 1000 bull genome project (http://www.1000bullgenomes.com) has identified candidate lethal mutations in genes such as *SMC2* (*structural maintenance of chromosomes*) and *COL2A1* (*collagen, type II, alpha 1*) [34, 35]. Interestingly, some of these deleterious variants are segregating at a significant frequency in the populations and contribute to negative genetic trends in fertility traits, and also likely affect the efficiency of in vitro embryo production.

Further advances in sequencing technologies include the development of long-read sequencing, yielding 20-kb sequence reads, which will improve existing livestock reference genome sequences which are still far from perfect. Once sequence data is available from a sufficient number of animals to define haplotypes in the population, it will be possible to predict high-density SNP genotypes from low-density data by “imputation”, indeed prediction of whole-genome sequence from high-density data will be possible. This high-resolution genome data will make the identification of the variants responsible for phenotypic variation more rapid. Molecular breeding using high-density SNP and even genome sequence data promises to be a game changer, resulting in faster and more efficient genetic selection. In spite of the decreasing costs, whole-genome sequencing is still 5- to 10-time more expensive than SNP genotyping and, at low-sequence coverage, it gives low-confidence genotype calls due to allelic drop-out in low-coverage genomic regions. Whole-genome sequencing could be expected to replace other genotyping methods in the near future as costs fall further.

### Functional genomics

#### Genome editing

Methods to introduce genetic variation into the genomes of animals have been used for many years, with the first transgenic mouse produced in 1981 [36]. However, the early transgenic approaches were unpredictable, unrepeatable [37], and invariably resulted in the insertion of the exogenous DNA into the host genome at multiple sites and in multiple copies. In some cases, this led to disruption of gene function, undesired ectopic expression that was difficult to control, and over- or under-expression of the inserted gene. Very recently methods for site-specific genetic modification have become available, and are now routinely used in research. These site-specific modifications are achieved by targeted cleavage of DNA and homologous recombination using zinc finger nucleases (ZFN), which are chimeric molecules, composed of a nuclease domain and specifically-designed DNA-recognition domains [38–40].

A more efficient molecular tool for genome editing is the clustered regularly interspaced short palindromic repeats (CRISPR)-associated 9 (Cas9) system. CRISPR/Cas9 uses short, single-guide RNA (sgRNA) to recognize target sequences in the DNA for Cas9 nuclease cleavage to facilitate editing [41]. A further development is the transcription activator-like effector nucleases (TALEN) genome editing system. These are novel fusion proteins that originate from plant pathogens in the bacterial genus *Xanthomonas* and contain DNA-binding motifs, which, when coupled to the *FokI* nuclease, create efficient gene-editing tools [42–44]. The TALEN and CRISPR/Cas9 systems are easier to engineer and more reliable than the use of zinc finger nucleases [45]. In the presence of single- or double-stranded DNA homologous to the target sequence, these systems can be used to introduce precise, targeted changes into the genome which may be deletions or additions of a few base pairs. It is also possible to introduce, delete or invert sequences of the genome that range in size from a few to several hundred nucleotides. It is even possible to target multiple genomic sites simultaneously and therefore modify several genes controlling complex traits, although the efficiency of multiple edits is still low.

Genome editing has the potential to accelerate genetic improvement of farm animals, by moving existing variations among populations, which up to now has been achieved by introgression through cross-breeding and successive rounds of back-crossing. In contrast, to this lengthy introgression process, genome editing can move alleles into specific genetic backgrounds in one generation.

The effective application of genome editing requires improvements in in vitro embryo methods, to provide large numbers of oocytes and increased efficiency of in vitro embryo production. Oocytes can be obtained by OPU and in vitro fertilisation followed by microinjection of the zygotes and then in vitro culture until a transferable embryo stage is reached, typically the blastocyst stage [41]. Improvements will include the production of oocytes from young females for a range of farm animal species using JIVET, which has been used successfully to create large numbers of viable embryos, but currently only in sheep. Improvements in in vitro culture techniques are also required to ensure correct embryo development and maximise the number of live progeny produced.

#### Biotech for breeding

Genome editing has many applications for advanced breeding, from repairing defective genes, such as recessive lethal or heritable disease variations in high genetic merit sires, to the introduction of genes that have a major effect on commercially important traits, such as resistance to disease and stress or polledness. An example application is improving heat tolerance in European breeds for production in tropical environments. The *SLICK* mutation, which was discovered in the Senepol Caribbean cattle breed [46], improves heat tolerance since it is associated with short hair and increased sweating. The SLICK phenotype is under the control of a single gene [46]. There are several mutations in the *prolactine receptor* gene that cause the SLICK phenotype, one of which is a premature stop codon caused by a frameshift mutation [47]. Introgression of this mutation in different breeds with recurrent backcross designs is possible, but is slow, since it requires several generations of backcrosses to regain the genetic qualities of the recipient breed. Introduction of the *SLICK* variations into a breed could be achieved in a single generation by gene editing. The ability to get a rapid response by the introduction of alleles that help animals to adapt to new environments is important in the face of rapid climate changes that have been predicted.

### Interactions between functional genomics, reproductive biotechnologies and breeding

Early developmental stages of in vitro produced embryos, up to blastocyst implantation, are associated with a high rate of failure. In sheep embryos, the period between day 20 and 30, during which there is the first functional interaction between the uterus and the extra-embryonic tissues leading to vascularisation, is a critical period. The success of this conceptus—mother interaction is affected by the epigenetic programming of the embryo, which in turn is affected by in vitro culture conditions. Comparison of the development of 89 naturally-conceived ovine conceptuses with 84 embryos produced in vitro revealed that a “foetal selection window”, specifically between day 24 and 26, is the period during which an epigenetically-compromised conceptus is most likely to die [48]. To better understand this phenomenon, we have analysed the gross morphology, histology and gene expression profiles of the placenta from naturally-conceived and in vitro produced embryos. Our results showed that in vitro produced conceptuses have defective cardiovascular development and more frequent haemorrhages because of impaired blood vessel development and integrity. These defects are associated with a significant down-regulation of the expression of vascular and angiogenetic factors (*FGF2*, *ANG2*, *TIE2* and *HOXA13)* detected in the placenta. Although these abnormalities in in vitro produced conceptuses were not necessarily lethal [49], there are clear deleterious implications for the lambs born from IVF procedures. Similar effects have been described, by others, for sheep IVF models [50], and similar placental anomalies have been observed for in vitro produced bovine embryos [51, 52].

It is likely that abnormalities associated with in vitro protocols have a common cause and may be due to epigenetic errors that accumulate during early development. These errors influence the establishment of the pregnancy, maternal-embryo communication and foetal development. Our work identified dysfunction of the DNA methylation machinery in the chorio-allantoic placenta from in vitro produced embryos [45]. During normal development, methylation of the gametic DNA is essentially lost shortly after fertilisation, and is then systematically re-established during early embryonic development, to be completely re-established by the blastocyst stage. In mammals, there are three major DNA methyltransferases, DNMT3a and b, which are involved in *de novo* methylation of DNA after embryo implantation, and DNMT1, which is necessary for the maintenance of established methylation patterns. A reduced DNMT1 activity may affect subsequent developmental processes. DNMT1 and cofactors (HDAC2, PCNA, DMAP1 and UHRF1) are significantly down-regulated in the placenta of in vitro produced embryos at very early developmental stages (day 20). As a consequence, expression of developmentally-important imprinted genes (*IGF2*, *H19*, *PEG1/MEST* and *CDKN1C*) are down-regulated. The methylation status of the maternal transcription factor H19 was also perturbed. These findings are in line with previous observations that epigenetic defects in the placenta result from in vitro embryo techniques, which are associated with growth arrest of the developing foetuses [53–55]. The expression profiles of imprinted genes varied among samples in our study and differ across studies [56, 57]. This suggests that these epigenetic defects arise from stochastic processes.

Impairment of one-carbon metabolism (OCM) could be one of the factors affecting the methylation mechanisms that underlie the observed developmental defects. OCM includes a series of biochemical reactions that are involved in the transfer one-carbon groups, and thus have a critical role in establishing and maintaining DNA methylation patterns (methyl or CH3 groups) [58]. Early pre-implantation development is characterized first by active, then passive genome demethylation during subsequent cleavages, followed by an increase in global DNA methylation. This methylation is associated with the control of gene expression during early differentiation and the formation of the inner cell mass and trophoblast at the blastocyst stage [59]. Hence, an impaired OCM metabolic pathway, and a deficiency in associated cofactors during in vitro early embryonic development, will result in epigenetic defects and early embryonic developmental abnormalities. These abnormalities include pregnancy loss, neural tube defects, intrauterine growth retardation, abnormal foetal brain development, and impaired cardiovascular development in foetuses [60, 61]. In addition to these early effects, impaired OCM can also have long-term consequences into post-natal life, such as metabolic diseases and impaired cognitive and motor function [61, 62].

Cobalamin (also known as vitamin B12), which is one of the main cofactors involved in OCM, is not present in the commonly used culture media for in vitro embryo production. Cobalamin supplementation during in vitro maturation of sheep oocytes has been shown to positively affect their developmental competence and the subsequent DNA methylation profile at the blastocyst stage [63], presumably through the OCM pathway. Cobalamin supplementation of the culture medium during in vitro fertilisation of oocytes also resulted in improved placental vascularisation. The positive effect of cobalamin addition to culture medium appears to act at the molecular level by increasing expression of DNA methyltransferases, thus correcting epigenetic mis-programming and preventing vascular defects. Our data suggest that simple improvements to the culture media by the inclusion of factors, including cobalamin, are likely to increase the efficiency and reliability of in vitro embryo production and reduce developmental abnormalities [64].

## Conclusions

There are potential benefits and synergies between genomic and reproductive technologies for sheep and cattle to enhance breeding programs. Advanced female reproductive technologies, such as MOET and JIVET [65] can increase the female contribution to the rate of genetic progress. Combining functional genetics and embryology to understand the epigenetic and gene expression effects of in vitro manipulation of gametes and embryos will contribute to optimising in vitro procedures; moreover, in vitro embryo biotechnologies, coupled with genome editing techniques, could provide the fittest phenotypes to withstand global climate changes [66]. Classically, modifications of protocols require validation through the production of offspring, which is time-consuming and expensive. However, molecular characterisation of in vitro produced embryos can provide information on the effects of changes in protocols, and indicate if these are beneficial or not. To conclude, improved in vitro embryo production is fundamental to enhance the genetic improvement of farm animals and the successful application of revolutionary techniques such as genome editing.
